# Super learner analysis of real‐time electronically monitored adherence to antiretroviral therapy under constrained optimization and comparison to non‐differentiated care approaches for persons living with HIV in rural Uganda

**DOI:** 10.1002/jia2.25467

**Published:** 2020-03-23

**Authors:** Alejandra E Benitez, Nicholas Musinguzi, David R Bangsberg, Mwebesa B Bwana, Conrad Muzoora, Peter W Hunt, Jeffrey N Martin, Jessica E Haberer, Maya L Petersen

**Affiliations:** ^1^ Division of Biostatistics School of Public Health University of California Berkeley Berkeley CA USA; ^2^ Global Health Collaborative Mbarara University of Science and Technology Mbarara Uganda; ^3^ Oregon Health & Science University‐Portland State University School of Public Health Portland OR USA; ^4^ Department of Internal Medicine Mbarara University of Science & Technology Mbarara Uganda; ^5^ Division of Experimental Medicine University of California San Francisco San Francisco CA USA; ^6^ Department of Epidemiology and Biostatistics University of California San Francisco San Francisco CA USA; ^7^ Massachusetts General Hospital Center for Global Health Boston MA USA; ^8^ Harvard Medical School Boston MA USA

**Keywords:** adherence, machine learning, real‐time adherence monitoring, viral load monitoring, virologic failure, viraemia

## Abstract

**Introduction:**

Real‐time electronic adherence monitoring (EAM) systems could inform on‐going risk assessment for HIV viraemia and be used to personalize viral load testing schedules. We evaluated the potential of real‐time EAM (transferred via cellular signal) and standard EAM (downloaded via USB cable) in rural Uganda to inform individually differentiated viral load testing strategies by applying machine learning approaches.

**Methods:**

We evaluated an observational cohort of persons living with HIV and treated with antiretroviral therapy (ART) who were monitored longitudinally with standard EAM from 2005 to 2011 and real‐time EAM from 2011 to 2015. Super learner, an ensemble machine learning method, was used to develop a tool for targeting viral load testing to detect viraemia (>1000 copies/ml) based on clinical (CD4 count, ART regimen), viral load and demographic data, together with EAM‐based adherence. Using sample‐splitting (cross‐validation), we evaluated area under the receiver operating characteristic curve (cvAUC), potential for EAM data to selectively defer viral load tests while minimizing delays in viraemia detection, and performance compared to WHO‐recommended testing schedules.

**Results:**

In total, 443 persons (1801 person‐years) and 485 persons (930 person‐years) contributed to standard and real‐time EAM analyses respectively. In the 2011 to 2015 dataset, addition of real‐time EAM (cvAUC: 0.88; 95% CI: 0.83, 0.93) significantly improved prediction compared to clinical/demographic data alone (cvAUC: 0.78; 95% CI: 0.72, 0.86; *p* = 0.03). In the 2005 to 2011 dataset, addition of standard EAM (cvAUC: 0.77; 95% CI: 0.72, 0.81) did not significantly improve prediction compared to clinical/demographic data alone (cvAUC: 0.70; 95% CI: 0.64, 0.76; *p* = 0.08). A hypothetical testing strategy using real‐time EAM to guide deferral of viral load tests would have reduced the number of tests by 32% while detecting 87% of viraemia cases without delay. By comparison, the WHO‐recommended testing schedule would have reduced the number of tests by 69%, but resulted in delayed detection of viraemia a mean of 74 days for 84% of individuals with viraemia. Similar rules derived from standard EAM also resulted in potential testing frequency reductions.

**Conclusions:**

Our machine learning approach demonstrates potential for combining EAM data with other clinical measures to develop a selective testing rule that reduces number of viral load tests ordered, while still identifying those at highest risk for viraemia.

## 1 Introduction

World Health Organization (WHO) guidelines now recommend antiretroviral treatment (ART) for all persons living with HIV, the majority of whom live in resource‐limited settings 1, 2. International consensus is increasing that effectively implementing universal treatment will require a differentiated care strategy, with the intensity of clinical follow‐up and monitoring varying based on individual patient need 3. In particular, the WHO now recommends that stable patients have plasma HIV RNA levels (viral loads) monitored less frequently than the quarterly monitoring previously recommended for all patients 2.

While decreasing monitoring frequency for stable patients can reduce costs for treatment programmes and patients, it remains unclear how to most effectively identify patients in need of more frequent monitoring. Delayed detection of adherence lapses and ongoing detectable viral replication (viraemia) can harm patient health, contribute to viral resistance and increase the risk of HIV transmission 4, 5, 6, 7. Strategies for tailoring monitoring intensity based on evolving metrics of patient risk for viraemia are needed to optimize both the impact and the cost‐effectiveness of differentiated ART delivery systems 8.

Electronic adherence monitoring (EAM) systems, which record a time‐date stamp whenever a medication storage device is opened as a proxy for medication ingestion, provide data to potentially inform such strategies. EAM systems could be used in combination with clinical data to identify patients at increased risk of viraemia, triggering both additional viral load monitoring to detect viraemia and adherence interventions to prevent it. EAM data can now be accessed in real‐time through cellular networks. The costs of this technology are falling 9, 10; however, the extent to which EAM systems can inform differentiated care decisions remains unclear.

EAM data can be summarized with many possible adherence metrics, including the proportion of prescribed doses for which an event is recorded, timing of device openings, and duration and frequency of lapses in openings. How best to select among these metrics and combine them with clinical data (such as duration of viral suppression and pre‐ART CD4^+^ T‐cell count) to assess the risk of viraemia is unknown. Modern machine learning approaches address this challenge by developing flexible and complex syntheses of EAM and clinical data to predict viraemia more accurately. Analysis of standard EAM data (stored on a device, but not available in real‐time) from HIV patients in the United States demonstrated that machine learning can improve the prediction and classification of viraemia 11. However, this approach has yet to be evaluated using either real‐time EAM data, which may differ in patient use and/or accuracy compared to standard EAM, or in a resource‐limited setting, where individual, immunological and virologic factors may differ from resource‐rich settings 12, 13, 14, 15.

We used machine learning methods to analyse standard and real‐time EAM data from an observational cohort of persons living with HIV in rural Uganda, and evaluated the added value of EAM technologies to predict viraemia, beyond the information provided by standard clinical and demographic data. We further assessed the potential for real‐time EAM data to effectively differentiate viral load testing frequency while minimizing delays in viraemia detection.

## 2 Methods

### 2.1 Study population

We analysed data from the Uganda AIDS Rural Treatment Outcome (UARTO) study (NCT01596322), an observational cohort of adults (>18 years) living with HIV who initiated ART in Mbarara, Uganda between 2005 and 2015. Participants lived within 60km of the Mbarara Regional Referral Hospital Immune Suppression Syndrome Clinic, which provides free ART in the region. To be eligible for real‐time monitoring, participants required a cellular signal at home 16.

### 2.2 Measures

ART adherence was monitored between 2005 and 2011 using standard electronic pill bottles (the Medication Event Monitoring System [MEMS], West Rock, Switzerland), from which time‐date stamps recording each device opening were downloaded onto a laptop computer via a USB cable during monthly home visits. From 2011 to 2015, adherence was measured using a real‐time electronic monitor that transmitted device opening data to a web‐based server using cellular networks (Wisepill; Wisepill Technologies, Cape Town, South Africa) 10. Lapses in device openings >48 hours detected using real‐time monitoring triggered a home visit to determine the cause of lapse. Participants were enrolled through 2012; thus, some participants were monitored with both device types.

Viral loads and CD4^+^ T‐cell counts were measured approximately quarterly, according to research protocol; after 2011, additional viral load measures were administered during home visits following adherence interruptions detected during real‐time monitoring. Viraemia was defined as a single viral load >1000 copies/mL, a threshold chosen to match WHO guidelines and minimize “blips” (temporary, low‐level increases in viral load) 17.

### 2.3 Statistical methods

#### 2.3.1 Risk score development

We used an ensemble machine learning method to build prediction models for viraemia. Viral loads measured ≥90 days after ART initiation were included as outcomes. Because detection of viraemia could affect both subsequent viral non‐suppression and monitoring, viral loads occurring after first detection of viraemia were censored. Risk scores were constructed separately for participants followed with standard versus real‐time EAM. Several candidate predictor sets were considered (Data S1).

1. “Clinical” predictors included age, biological sex, CD4^+ ^T‐cell counts before and after ART initiation, and ART regimen (drugs, regimen changes, prescribed dosing interval and time since initiation).
2. “EAM + Clinical” predictors augmented the clinical predictors with additional EAM data. Candidate EAM features were evaluated over a range of periods preceding each viral load measurement (from seven to three hundred and sixty‐five days). For each of these periods, we calculated daily adherence (number of EAM events/total number of prescribed doses), variance of daily adherence, minimum adherence, number and duration of interruptions in events and variability in timing between recorded events. To evaluate the extent to which ongoing CD4^+^ monitoring improved prediction in the context of EAM, we also considered a predictor set excluding post‐ART initiation CD4^+^ counts.
3. “Full EAM” predictors augmented clinical and EAM predictors with viral load data, including either (a) viral load at ART initiation only or (b) all time‐varying viral load data.

Predictor variables missing a measurement were imputed using last measured value, with time since last measurement included as a predictor. Tests with missing predictor values (after imputation) or occurring after a > 400‐day lapse in testing (threshold chosen based on the distribution of lapses in monitoring) were excluded.

Super learner, an ensemble method which combines several “candidate” machine learning algorithms using internal cross‐validation, was used to construct a prediction model for viraemia using each predictor set 18. Leaving aside each fold in turn as validation data, candidate prediction algorithms were fit on the remaining 9/10ths of the data. Validation data were then used to select the convex combination of algorithms that maximized the rate of negative prediction under a constraint to maintain sensitivity above 93% (constraint raised to 95% in sensitivity analyses), together with a corresponding cutoff for positive classification 19. This threshold was chosen to improve rate of negative prediction while maintaining a clinically acceptable sensitivity. The following algorithms were included as candidates: gradient boosting machine 20, random forests 21, Bayes generalized linear models 22 and elastic net 23, each with and without a dimension reduction based on marginal correlation with the outcome.

#### 2.3.2 Performance

An additional layer of cross‐validation was used to evaluate the performance of the prediction models by calculating performance metrics in each independent validation set and averaging. Individual participants were stratified based on viral suppression status before sample‐splitting to ensure that each fold had a similar class balance; all sample splitting respected the individual as the unit of independence. While the machine learning algorithm aimed to optimize differentiated testing rather than to accurately predict the full range of risk, as global measures of performance we plotted cross‐validated receiver operating characteristic (cvROC) curves and calculated area under the cvROC curve (cvAUC). Differences in cvAUC for distinct predictor sets were tested using the influence function of the cvAUC to derive a z‐test 24. We also calculated net reclassification improvement, and plotted calibration and conducted the Hosmer‐Lemeshow test (Data S2).

We then evaluated the potential of each of the candidate predictor sets to reduce the viral load testing frequency and increase the yield when combined with a selected cutoff chosen in the corresponding training set (to accurately assess performance of the learned testing rule versus the risk predictor alone on independent data). Specifically, we calculated the cross‐validated rate of negative prediction (cvRNP; proportion of viral load tests that would have been avoided because predicted risk of viraemia was below the cutoff), “number needed to screen” (cvNNS; number of viral load tests with predicted risk above the cutoff/ the number of viraemia cases with predicted risk above the cutoff), empirical sensitivity (cvSens; proportion of viraemia cases with predicted risk above the cutoff), false‐positive rate (cvFPR; proportion of non‐viraemia cases with predicted risk above the cutoff) and the precision (cvPPV; number of viraemia cases with risk score above the cutoff/ number of risk scores above the cutoff).

We further evaluated cross‐validated performance of three hypothetical strategies for viral load monitoring: (1) A “3‐month” schedule, in which viral load was measured every three months (a reference for comparison that, while not realistic in many settings, was available for the study population); (2) A “WHO” schedule: in which viral load was measured at six and twelve months after ART initiation and annually thereafter, as recommended for stable patients 2 (a schedule now routine care in Uganda); and, (3) An “EAM”‐based differentiated monitoring strategy, in which the WHO schedule was augmented with additional viral load tests on dates that the predicted risk of viraemia exceeded the cutoff (using all predictors except viral loads and restricting EAM‐triggered tests to dates without missing predictors). For each strategy, we calculated the monitoring rate (tests ordered per person‐year), sensitivity, NNS, FPR and delay to viraemia detection relative to observed date of first detection under research protocol (corresponding to maximal but unrealistic testing frequency). To do so, we assumed that omitting an observed test would not change future adherence or viraemia and that if an observed viraemic test were omitted, viraemia would still be present and would not be detected until the next test.

Analyses were performed using R version 3.5.0 25, and packages SuperLearner 26, xgboost 27, 28, bartMachine 27, 28, glmnet 29, arm 30, ROCR 31 and predictABEL 32.

### 2.4 Ethics

The Mbarara University of Science and Technology, the Uganda National Council for Science and Technology, Partners Healthcare, and the University of San Francisco, California ethical review boards approved this study. All participants provided written informed consent.

## 3 Results

### 3.1 Sample characteristics

A total of 443 participants were monitored with standard EAM for a median of 4.6 years (IQR: 2.5 to 5.6) and contributed 5922 viral load results as outcomes in the standard EAM analysis dataset; 485 participants were monitored with real‐time EAM for a median of 2.2 years (IQR: 1.4 to 2.5) and contributed 2834 viral load results as outcomes in the real‐time EAM analysis dataset (Table 1). Of real‐time EAM users, 243 had been monitored with standard EAM before initiating real‐time monitoring. Between 2005 and 2011, 86 of the 443 participants (19%) monitored with standard EAM experienced viraemia (86/5923 tests, 1.5%). Between 2011 and 2015, 45 of the 485 participants (9.3%) monitored in real‐time experienced viraemia (45/2834 tests, 1.6%).

**Table 1:** Baseline^a^ characteristics of the study population

	Standard EAM (N = 443)	Real‐time EAM (N = 485)
Woman	N = 307 (69.3%)	N = 345 (71.1%)
Age (years)	median 35 (IQR: 30 to 39)	median 33 (IQR: 27 to 40)
Follow‐up time (years)	Median 4.6 (IQR: 2.5 to 5.6)	Median 2.2 (IQR: 1.4 to 2.5)
CD4^+^ T‐cell count (cells/mm^3^) at ART Initiation	Median 135 (IQR: 78 to 202)	Median 200 (IQR: 111 to 317)
NNRTI at baseline	N = 440 (99.3%)	N = 447 (92.2%)
Efavirenz	N = 57 (13%)	N = 228 (51%)
Nevirapine	N = 383 (87%)	N = 219 (49%)
Plasma HIV RNA level (viral load) (copies/ml) at ART Initiation	Median 113,888 (IQR: 39,789 to 343,272)	Median 94,041 (IQR: 30,631 to 299,705)
Total days from ART initiation to baseline^a^	Median 168 (IQR: 164 to 175)	Median 112 (IQR: 107 to 120)^b^
Initiated ART within 120 days prior to first EAM monitoring	N = 72 (16%)	N = 187 (39%)
Viral load tests included as outcomes	5922 tests^c^, ^a^	2834 tests^d^
Participants Continuing Monitoring from Standard EAM	NA	N = 243

Among HIV‐infected adults followed with electronic adherence monitoring using either standard or real‐time devices following ART initiation in Uganda. Missing for standard EAM Users: Baseline Viral Load (n = 8; 1.8%), sex (n = 5; 1.1%), age (n = 5; 1.1%). Missing for real‐time Users: Baseline Viral Load (n = 12; 2.5%), Baseline CD4 (n = 5; 1%). IQR. interquartile range; NNRTI, non‐nucleoside reverse transcriptase inhibitor.

^a^ Baseline: first viral load test while using electronic adherence monitoring;

^b^ estimate is among participants continuing after being monitored by standard EAM;

^c^ 15 tests (0.2%) excluded due to a greater than 400‐day lapse in testing. An additional 300 tests (5%) excluded from machine learning training set, but not from evaluation of performance, due to no adherence data in at least 180 days or missing predictor data;

^d^ 389 tests (12%) excluded due to a greater than a 400‐day lapse in testing; 98% of these occurred during the final three months of study follow‐up. An additional 168 tests (6%) excluded from machine learning training set, but not from evaluation of performance, due to no adherence data in at least 180 days or missing predictor data.

Consecutive viral load tests were a median of 105 days (IQR: 97 to 114) and 115 days (IQR: 97 to 178) apart for standard and real‐time EAM users respectively; 4% of tests under standard EAM monitoring and 25% of tests under real‐time EAM monitoring were administered <60 days since the prior test; under real‐time monitoring 36% of these were preceded by a 48‐hour interruption. EAM data were measured a median of 52 days (IQR: 32 to 78) and 84 days (IQR: 75 to 88) of the 90 days preceding a viral load test in the standard and real‐time EAM datasets respectively. During standard monitoring, during the 90 days preceding a viral load test median average adherence was 89% (IQR: 75% to 96%) with a median of 2 treatment interruptions ≥24 hours (IQR: 1 to 4). During real‐time monitoring, during the 90 days preceding a viral load test median average adherence was 93% (IQR: 86% to 97%) with a median of 3 interruptions of ≥24 hours (IQR: 1 to 8).

#### 3.1.1 Contribution of EAM to machine learning‐based prediction of viraemia

**Figure 1:**
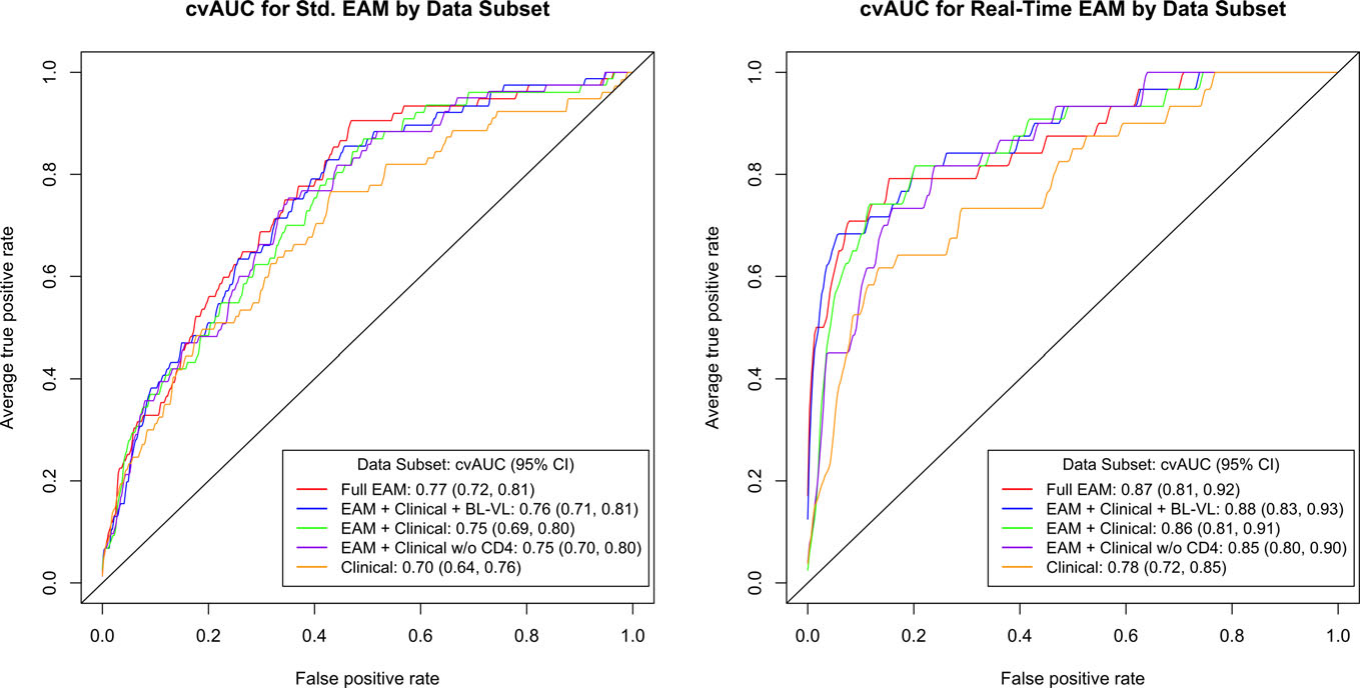
Cross‐validated (10‐Fold) ROC Curve for super learner prediction of viraemia using 5 predictor sets (Data S1). Viraemia defined as HIV RNA level >1000 copies per mL. blVL, Baseline Viral load; EAM, Electronic Adherence Monitoring; VL, viral load.

Super learning applied to standard EAM, clinical and demographic data (“Full EAM” predictors) yielded a cvAUC for viraemia of 0.77 (95% CI: 0.72, 0.81), non‐significantly (*p* = 0.08) higher than the cvAUC of 0.70 (95% CI: 0.64, 0.76) achieved using clinical predictors alone (Figure 1, Table 2). Addition of standard EAM data without viral loads to the clinical predictor set resulted in a modest but non‐significant (*p* = 0.27) increase in the cvAUC (0.75; 95% CI: 0.69, 0.80). Additional inclusion of baseline viral load or removal of CD4^+^ T‐cell count from the predictor set had a minimal impact on cvAUC.

**Table 2:** Cross‐validated AUROC, cross‐validated rate of negative prediction, cross‐validated sensitivity and cross‐validated number needed to screen

	Full EAM	Clinical + EAM + blVL	Clinical + EAM	Clinical + EAM, no CD4	Clinical
Std. EAM					
cvAUC (95% CI)	0.77 (0.72, 0.81)	0.76 (0.71, 0.81)	0.75 (0.69, 0.80)	0.75 (0.70, 0.80)	0.70 (0.64, 0.76)
cvRNP	0.31	0.29	0.26	0.25	0.15
cvSens	0.93	0.95	0.96	0.96	0.94
cvNNS	51	52	54	55	62
cvFPR	0.69	0.71	0.74	0.75	0.85
cvPPV	0.02	0.02	0.02	0.02	0.02
Real‐time EAM					
cvAUC	0.87 (0.81, 0.92)	0.88 (0.83, 0.93)	0.86 (0.81, 0.91)	0.85 (0.80, 0.90)	0.78 (0.72, 0.85)
cvRNP	0.38	0.37	0.47	0.39	0.28
cvSens	0.91	0.9	0.88	0.9	0.93
cvNNS	48	47	41	46	56
cvFPR	0.62	0.63	0.53	0.60	0.72
cvPPV	0.02	0.02	0.02	0.02	0.02

AUROC, area under the ROC curve; cvAUC, cross‐validated area under ROC curve; cvFPR; cross‐validated false positive rate; cvNNS, cross‐validated number needed to screen; cvPPV, cross‐validated positive predictive value (precision); cvRNP, cross‐validated rate of negative prediction (proportion of tests avoided); cvSens, cross‐validated sensitivity; EAM, Electronic Adherence Monitoring; VL, viral load; blVL, Baseline Viral load.

Super learning applied to clinical predictors alone achieved a cvAUC in the real‐time EAM data set of 0.78 (95% CI: 0.72, 0.85). In contrast to the modest improvement in performance achieved with standard EAM data, addition of baseline viral load and real‐time EAM predictors to the clinical predictors resulted in a significant (*p* = 0.03) improvement in the cvAUC (0.88 95% CI: 0.83, 0.93). Addition of real‐time EAM data alone to the clinical predictors resulted in moderate improvement in the cvAUC (*p* = 0.06) (0.86 95% CI: 0.81, 0.91). Removal of time‐varying CD4 count from the “EAM + Clinical” set had no impact on the cvAUC, suggesting little incremental gain in prediction of viraemia from this measure (Table 2). Comparisons of alternative predictor sets based on net reclassification improvement were qualitatively similar for both standard and real‐time EAM; for EAM + Clinical predictor set (used in the EAM‐based testing strategy) the Hosmer‐Lemeshow test supported adequate calibration (Data S2).

#### 3.1.2 Potential performance of a EAM‐guided differentiated monitoring strategy

We evaluated hypothetical rules for triggering viral load tests based on combining the machine learning risk score with a cutoff above which a viral load test would be ordered. The cutoff was chosen to meet the sensitivity‐constrained criteria (Table 2)*.* When trained on standard EAM data, the super learner attained a cross‐validated sensitivity of 93% to 96% and rate of negative prediction of 25% to 31%. When trained on real‐time EAM data, super learner attained a cross‐validated sensitivity of 88% to 91% and rate of negative prediction of 37% to 47%.

Finally, we compared the performance of two non‐differentiated strategies to a modified version of the machine learning classification procedure as described above (Table 3). When based on standard EAM data, the “3‐month” schedule would have reduced the number of tests ordered by 3% and would have delayed detection of 2% of observed viraemia cases, for an average delay in detection among all viraemia cases of one day. In contrast, the “WHO” schedule would have reduced the number of tests ordered by 77%, but would have resulted in the delayed detection of 67% of viraemia cases, with an average delay in detection of 61 days. Finally, the “EAM” machine learning approach could have reduced the total number of viral load tests ordered by 24% while delaying detection of only 9% of viraemia cases, with an average delay in detection of nine days. Under “3‐month,” “WHO” and “EAM” schedules, 97%, 22% and 76% of non‐viraemic cases would have received a test respectively.

**Table 3:** Performance of “3‐Month,” “WHO” and “EAM” testing rules

	Standard (EAM, CD4, w/o VL)				Real‐time (EAM, CD4, w/o VL)			
	3‐month	WHO	EAM^c^, ^a^	Obs	3‐month	WHO	EAM^c^, ^a^	Obs
RNP	0.03	0.77	0.24	0	0.19	0.69	0.32	0
1‐Sensitivity	0.02	0.67	0.09	0	0.16	0.84	0.13	0
No. Tests Total	5728	1333	4518	5922	2304	886	1928	2834
FPR	0.97	0.22	0.76	0	0.81	0.32	0.68	0
Mean, median delay time among undetected viraemia cases, days (IQR)	42, 43 (37 to 48)	91, 96 (88 to 101)	97, 96 (89 to 97)	NA	49, 48 (31 to 64)	88, 76 (68 to 107)	84, 74 (73 to 75)	NA
Mean, median delay time among all viraemia cases, days (IQR)	1, 0 (0 to 0)	61, 88 (0 to 97)	9, 0 (0 to 0)	NA	8, 0 (0 to 0)	74, 73 (62 to 97)	11, 0 (0 to 0)	NA

3‐month, Test every 3 mos; EAM, WHO schedule with additional testing if algorithm predicts high risk of viraemia; FPR, False positive rate; Obs, Observed testing schedule for performance reference; RNP, rate of negative prediction; WHO, Test 6 and 12 mos after ART initiation and yearly thereafter.

^a^ “EAM” performance metrics differ slightly from Table 2 due to testing schedule augmented by “WHO” schedule (See Methods).

Using the real‐time EAM data, the “3‐month” schedule would have avoided 19% of observed tests (a reduction relative to the observed schedule due to eliminating extra tests triggered by detected interruptions) and delayed detection of 16% of viraemia cases by eight days on average, while the “WHO” schedule would have reduced the number of tests by 69%, and delayed detection of 84% of viraemia cases by 74 days on average. In contrast, the “EAM” approach would have avoided 32% of all viral load tests, while delaying detection of 13% of viraemia cases with an average delay of 11 days. Under “3‐month,” “WHO” and “EAM” schedules, 81%, 32% and 68%, of non‐viraemic cases would have received a test respectively.

## 4 Discussion

Analysis of real‐time electronic adherence data using ensemble machine learning achieved excellent prediction of viraemia among HIV‐infected individuals treated with ART in rural Uganda (cvAUC of 0.88). Addition of real‐time EAM and viral load data to basic demographic and clinical data significantly improved prediction of viraemia, indicating potential value added by this technology. However, further addition of post‐ART CD4^+^ T‐cell counts to the predictor set did not significantly improve global predictive performance (as assessed with cvAUC), supporting prior findings of the limited value of CD4 count for predicting viraemia 33, 34, 35.

Our results suggest that a testing strategy using real‐time EAM to decide when to order versus defer viral load testing could substantially reduce the number of viral load tests ordered (32% to 47% of observed tests avoided, depending on the strategy and availability of viral load), while still detecting most viraemia cases without additional delay. While some strategies incorporated baseline viral load, which may not be routinely available, the benefits of an EAM‐based differentiated testing approach were also substantial when viral loads were not used. By comparison, the testing schedule recommended by the WHO for stable patients, if deployed uniformly for all participants in our sample, would have reduced the number of tests ordered by 69%, but would have delayed detection of viraemia 74 days on average among individuals experiencing viraemia. Extended viraemia increases the risk of developing drug‐resistant virus, 36 and of onwards transmission of HIV infection 37 *.*

Super learning applied to standard EAM data, in combination with clinical and demographic data, was able to predict viraemia well (cvAUC of 0.77), and would have avoided 25% to 31% of observed viral load tests while detecting most viraemia cases without additional delay. In contrast, the improvement in prediction seen with addition of standard EAM data to clinical and demographic data was not statistically significant (*p* = 0.08). Differences in the “value‐added” of real‐time versus standard EAM may have been due to differences in participant characteristics or temporal trends – participants in the real‐time EAM cohort were followed more recently and had higher CD4^+^ T‐cells at ART initiation. Implementation of real‐time monitoring may also have provided better information to guide differentiated testing compared to standard monitoring by allowing for real‐time data quality improvements (e.g. identifying periods of device non‐use). Furthermore, interruptions in events could trigger additional tests during real‐time but not standard monitoring; thus, both the reference “observed” testing regime differed and the extra tests themselves may have changed adherence*.* Indeed, average adherence appeared to increase when participants were switched from standard to real‐time monitoring 10, and qualitative work supports a possible motivational effect of “being watched” 38, 39. However, the number of >24 hour interruptions was similar if not higher during real‐time monitoring. These sustained interruptions may be related to structural barriers to adherence (e.g. lack of transportation to pick up medication) that are not as amenable to changes in motivation and instead reflect circumstantial differences in the two monitoring periods.

Our study has limitations. First, we assumed that excluding observed tests would not have changed subsequent adherence, viraemia, or their relationship; to improve the plausibility of this assumption, we censored at first detected viraemia. However, less frequent monitoring might affect adherence by reducing positive feedback provided to participants. Second, use of a real‐time monitoring strategy to guide viral load testing would make possible not only targeted reduction in testing frequency, but also early adherence and testing interventions (beyond those implemented in the real‐time EAM study); the current analysis is conservative in the sense that it does not incorporate these additional potential benefits. Third, while estimates of the proportion of viral load tests that could be deferred under hypothetical testing strategies were based on independent data (via cross‐validation), accurate quantification of their uncertainty is an area of current work.

Finally, cost is an obvious concern when considering technology for clinical care. Cost‐effectiveness is beyond the scope of this analysis; however, a cost‐effectiveness analysis of potential ART adherence monitoring interventions in sub‐Saharan Africa found that an adherence monitoring‐based intervention could cost up to $50 per person‐year on ART while remaining cost‐effective, mainly driven by savings through effective differentiation of care 8. Current lower cost versions of real‐time adherence monitoring devices consistent with that threshold 40 are now available and are being tested for use in routine care (NCT03825952). Application of a previously developed machine learning tool does not require intensive computing resources 41, and the increasing use of smart phones globally 42 could make even real‐time updates to a machine learning algorithm feasible in the foreseeable future. Further, a differentiated care approach could be used to target use of these devices (e.g. in patients with self‐reported adherence challenges).

## 5 Conclusions

Evidence is increasing that differentiated care for HIV patients in resource‐limited settings is a cost‐effective intervention 3, 8. Real‐time EAM provides one possible tool to support a differentiated care strategy by making it possible to offer viral load testing and adherence interventions on an individualized schedule in response to evolving patient needs. Similar low‐cost, real‐time technology is being used in clinical care for tuberculosis and is being assessed for ART in Uganda 40. This technology allows providers to triage at‐risk patients. However, the informative real‐time data that rapidly accumulate through these devices may be difficult to interpret. Flexible algorithms with the capacity to leverage these data, such as those presented here, could be readily integrated into accessible software to address this issue.

In conclusion, our analysis suggests that real‐time electronic adherence data analysed with machine learning methods have the potential to achieve a more efficient targeted viral load monitoring strategy while maintaining high sensitivity for detection of viraemia. Future work should prioritize external validation of differentiated strategies in new settings, and implementation of these methods through software that aims to guide differentiated patient care. Our results provide an illustration of the utility of machine learning methods to better leverage complex data for precision medicine and public health.

## Competing interests

The authors have no conflicts of interest to disclose.

## Authors’ contributions

MLP, DRB, JEH and AEB conceived and planned the analysis. BMB, CM, DRB, PWH, JNM and JEH designed the parent study and oversaw study implementation and data collection. AEB implemented the analysis with guidance from MLP and support from NM. AEB and MLP wrote the initial version of the manuscript with significant input from JEH. All authors contributed to interpretation and writing and approve the submitted version of the manuscript.

## Supporting information


**Data S1.** Supplemental Material for Super Learning Analysis of Real‐Time Electronically Monitored Adherence.

**Data S2.** Supplemental Material (Part II) for Super Learning Analysis of Real‐Time Electronically Monitored Adherence.
