# Supplementary material for: Super learner analysis of real‐time electronically monitored adherence to antiretroviral therapy under constrained optimization and comparison to non‐differentiated care approaches for persons living with HIV in rural Uganda
Source: J Int AIDS Soc. 2020 Mar 23;23(3):e25467. doi: 10.1002/jia2.25467 (PMC7086301; doi:10.1002/jia2.25467)
Supplement: Supplementary file 1 — Data S1. Supplemental Material for Super Learning Analysis of Real‐Time Electronically Monitored Adherence. [file JIA2-23-e25467-s001.pdf]

1 Training Sets

Various training sets were created by merging all or subsets of the following data sources: demographic, baseline clinical, time-varying clinical, time-varying viral load data, ART and electronic adherence monitoring (EAM) summaries.

The following distinct training sets were considered (subsets of the predictor variables):

(a) All predictors.

(b) Remove time-varying viral load data.

(c) Remove time-varying viral load data, and baseline viral load.

(d) Remove time-varying viral load data, baseline viral load, and time-varying CD4.

(e) Remove time-varying viral load data, baseline viral load, and EAM.

2 Data Descriptions

2.1 Demographic / Baseline CD4

- **Age**: Patient age (years).
- **Sex**: Binary indicator of female gender.
- **Baseline CD4**: Last measured CD4 with date $\leq$ ART start date.

2.2 Time-varying CD4

- **Last CD4**: Last measured CD4 value.
- **Time Since CD4 Measured**: Number of days since CD4 measurement was recorded.
- **Nadir CD4**: min of all CD4s with date $\leq$ current viral load (VL) date.

2.3 Baseline Viral Load

Let Q_{pre} be the last VL test date prior to the first regimen start date.

- **Days between first regimen start date and** Q_{pre}: First regimen start date - Q_{pre} (days)
- **Time since** Q_{pre}: Current (row) date - Q_{pre} (days)

2.4 Time-varying Viral Load

- **Last Viral Load**: Previous VL result.
- **Last Viral Load Test at Limit**: Binary indicator of previous VL result at the limit of detection.
- **Time Since Last Viral Load Test**: Number of days since last VL test.
- **Previous VL > 400**: Number of previous VL tests that were $>$ 400 copies/ml, which occurred prior to current visit and $\geq$ 90 days after ART start date.

2.5 ART

- **Time On Study**: Days since start of EAM (either standard or real-time).
- **ARV Code**, binary indicator for each of the following ARV codes (21 total*): 3TC, ABC, ALU, ATR, ATZ, AZT, AZR, D4T, DUN, DUO, EFV, LAS, LPV, NVP, RAL, RTV, TDF, TLA, TLE, TRI, TRU
- **Drug Class**, binary indicator for each of the following drug classes: NRTI, PI, NNRTI, and Other
- **ARV Regimen**, binary indicator columns, where regimen varies over the unique ARV code combinations (53 total).
- **Med Class** for $X \in \{0, 1, 2, 4\}$: Regimen class* (no instances of type 3 or 5 in training data). Note that this is 0 only prior to ART start. These are binary indicator columns, but each row belongs to only one med class.
- **Num Drugs Current**: Number of unique ARV codes in current regimen.
- **Num Drug Classes Current**: Number of unique drug classes in current regimen.
- **New Drug Introduced**: Binary indicator of a new ARV code (new = 1). All visits prior to and including ARV start date are coded as 0.
- **New Regimen Introduced**: Binary indicator of a new ARV code based regimen (new = 1). All visits prior to and including ARV start date are coded as 0.
- **Num Regimen Changes**: Number of changes in ARV code-based regimens since ART start (change defined as: current ARV code != Prior ARV codes)
- **Num Med Classes Ever**: Number of unique med classes since ART start.
- **Num Drug Classes Ever**: Number of unique drug classes since ART start.
- **Num Drugs Ever**: Number of unique ARV codes ever used.
- **Daily Total Doses**: Number (0-10) of doses prescribed per day across all medications. (bid=2, qd=1)
- **Dose Frequency**: Maximum number (0-2) of daily doses among the unique medications. (bid=2, qd=1)
- **Any X** for X $\in$ {3TC, TDF, AZT, D4T, EFV, NVP, FTC}.*
- **Baseline Year**: Calendar year at ART start date.

*See the appendix for detailed ARV information.

2.6 EAM (Adherence Summaries)

- **Days Since Last Monitoring**: Current date − date of previous monitoring event.
- **Avg Adherence X** for $X \in \{7, 14, 30, 60, 90, 180, 365\}$: Average adherence over the last X days.
- **Avg Adherence VL**: Average adherence since the last VL test.
- **Days Monitored X** for $X \in \{7, 14, 30, 60, 90, 180, 365\}$: Number of days monitoring data exists over the last X days.
- **Min Adherence X, N** for $X \in \{7, 14, 30, 60, 90, 180, 365\}$ and $N \in \{2, 3, 4, 7, 14, 30\}$: Minimum adherence over the past X days using N-day sliding windows, such that $X > N$.
- **Min Adherence VL N** for $N \in \{2, 3, 4, 7, 14, 30\}$: Minimum adherence since the last VL test using N-day sliding windows.
- **Variance of Adherence X** for $X \in \{7, 14, 30, 60, 90, 180, 365\}$
- **Variance of Adherence VL**: Variance of adherence since the last VL test.
- **Num Interruptions X, Y** for $X \in \{7, 14, 30, 60, 90, 180, 365\}$ and $Y \in \{1, 2, 3, 4, 7, 10, 11, 12, ..., 19, 20, 25, 30\}$: Number of interruptions over the past X days, of at least Y days.
- **Num Interruptions VL Y** for $Y \in \{1, 2, 3, 4, 7, 10, 11, 12, ..., 19, 20, 25, 30\}$: Number of interruptions since the last VL test, of at least Y days.
- **Variance of inter-dosing interval in last X days** for $X \in \{7, 14, 30, 60, 90, 180, 365\}$: Variance of inter-dosing interval over the last X days.
- **Variance of Inter-Dosing intervaI**: Variance of inter-dosing interval since the last VL test.

3 Training Set: Predicting Failure

The training data sets are constructed from the original viral load (VL) test result data. Each row corresponds to a clinic visit with a non-missing viral load result. Each patient has multiple visits, so we treat the data as a pooled repeated measures data set. Rows are uniquely determined by patient ID and visit date. If the outcome is missing, the sample will be excluded. Additionally, visits for which the EAM data is missing or outdated (more than 180 days old) are excluded.

3.1 Outcome of interest: Binary indicator of viral failure

The binary outcome of interest is viral failure. We defined this is as:

- Failure := $VL > 1000$ copies/mL

3.1.1 Start Date

The start date is the first visit which is at least 90 days after ART start date, for which there is prior EAM data.

3.1.2 End Date

The end date is the earliest of: {date of first Failure, last visit}

3.2 Imputation

EAM variables were imputed using the most recent available value. For example, if avgAdh_7 and avgAdh_14 were missing, but avgAdh_30 was available, then both would be imputed with the avgAdh_30 value. Only EAM variables were imputed, and the remainder of the rows that still contained missing values in non-EAM columns were removed.

4 Appendix

medClass	Description
0	Has not yet started treatment
1	Any NNRTI and no PI
2	Any PI and no NNRTI
3	Both NNRTI and PI
4	All NRTI
5	Other

Table 1: Definition of medClass variable

Variable	Associated ARV codes
any3TC	x3TC, DUO, TLE, TRI
anyTDF	TDF, ATR, TLE, TRU
anyAZT	AZT, DUO, DUN
anyD4T	D4T, TRI
anyEFV	EFV, ATR
anyNVP	NVP, DUN, TRI
anyFTC	TRU, ATR

Table 2: Definition of anyX set of variables
