# Supplementary material for: Super learner analysis of real‐time electronically monitored adherence to antiretroviral therapy under constrained optimization and comparison to non‐differentiated care approaches for persons living with HIV in rural Uganda
Source: J Int AIDS Soc. 2020 Mar 23;23(3):e25467. doi: 10.1002/jia2.25467 (PMC7086301; doi:10.1002/jia2.25467)
Supplement: Supplementary file 2 — Data S2. Supplemental Material (Part II) for Super Learning Analysis of Real‐Time Electronically Monitored Adherence. [file JIA2-23-e25467-s002.pdf]

Supplemental Material (Part II) for Super Learning Analysis of Real-Time Electronically Monitored Adherence

In addition to testing the differences in cvAUC between data subsets using the standard error estimates based on the cvAUC's influence function, the change in classification performance of the super learner based risk predictor performance across sets of predictor variables was also tested using the net reclassification improvement (NRI) statistic. The NRI assesses a prediction model's classification performance relative to a reference model by quantifying the new model's changes in risk score assignment. Changes in risk classification occur when the prediction model being compared categorizes an individual to a higher or lower risk, relative to the reference model [1]. The NRI analysis led to results that were similar to those obtained when the change in cvAUC was tested using the influence function.

Table 1. Net Reclassification Improvement (NRI)

	E-A	E-B	E-C	E-D
Std. EAM.NRI	0.243	0.138	0.151	0.036
Std. EAM.NRI_pval	0.034	0.226	0.186	0.754
Real-time EAM.NRI	0.750	0.921	0.634	0.523
Real-time EAM.NRI_pval	0.000	0.000	0.000	0.002

Relative to Data Subset E (Clinical)
A; Full EAM
B; Clinical + EAM + blVL
C; Clinical + EAM
D; Clinical + EAM, no CD4

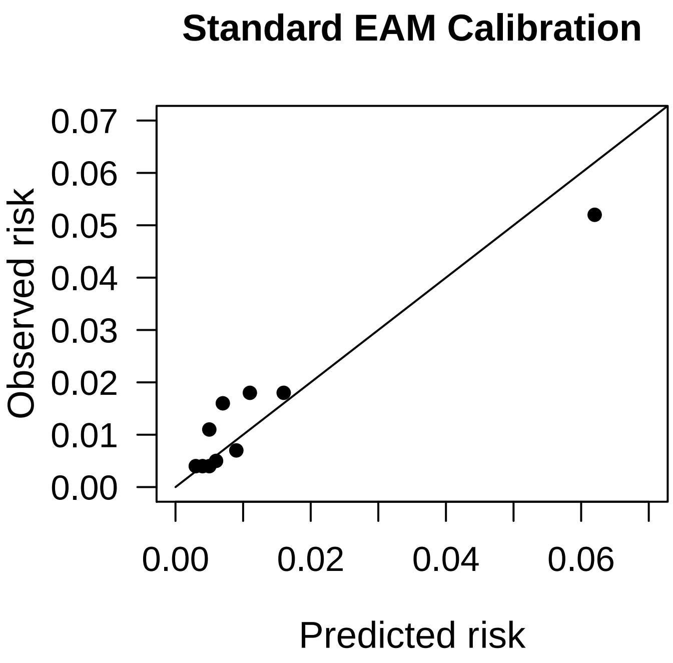

Figure 1: Calibration Plot for Standard EAM
Based on EAM, CD4, w/o VL
Among HIV-infected adults followed with standard EAM

Table 2: Hosmer-Lemeshow Test for Standard EAM

risk score decile	total obs.	mean pred.	mean obs.	pred. # cases	obs. # cases
[0.00159,0.00374)	563	0.003	0.004	1.81	2
[0.00374,0.00441)	562	0.004	0.004	2.30	2
[0.00441,0.00508)	562	0.005	0.004	2.66	2
[0.00508,0.00582)	562	0.005	0.011	3.05	6
[0.00582,0.00672)	562	0.006	0.005	3.52	3
[0.00672,0.00791)	563	0.007	0.016	4.10	9
[0.00791,0.00978)	562	0.009	0.007	4.91	4
[0.00978,0.01272)	562	0.011	0.018	6.26	10
[0.01272,0.02152)	562	0.016	0.018	9.04	10
[0.02152,0.60313]	562	0.062	0.052	34.92	29

Based on EAM, CD4, w/o VL
Using 10 risk score bins; Hosmer-Lemeshow goodness of fit p-value: 0.106
total obs.; total number of observations in risk decile
mean pred.; mean number of predicted viremic cases in risk decile
mean obs.; mean number of observed viremic cases predicted in risk decile
pred. # cases; predicted number of viremic cases in risk decile
obs. # cases; observed number of viremic cases in risk decile

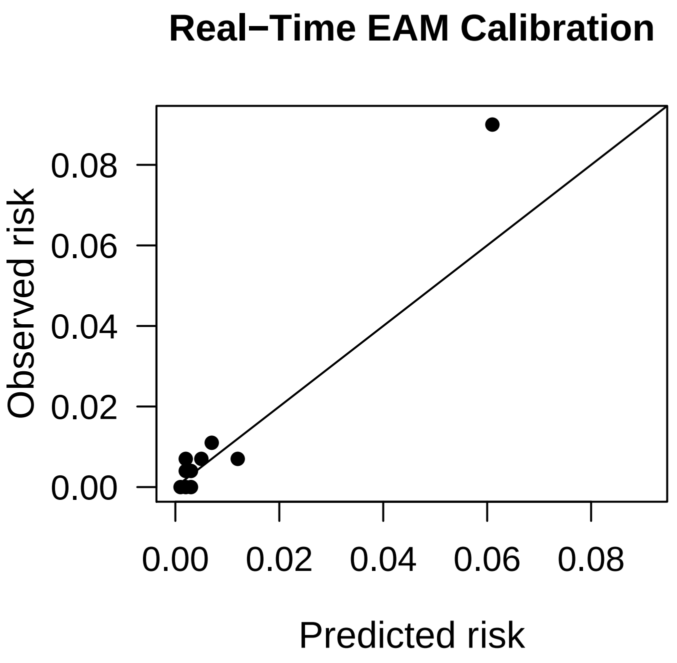

Figure 2: Calibration Plot for Real-time EAM
Based on EAM, CD4, w/o VL
Among HIV-infected adults followed with real-time EAM

Table 3: Hosmer-Lemeshow Test for Real-Time EAM

risk score decile	total obs.	mean pred.	mean obs.	pred. # cases	obs. # cases
[0.000932,0.00142)	267	0.001	0.000	0.33	0
[0.001420,0.00171)	267	0.002	0.004	0.42	1
[0.001707,0.00205)	266	0.002	0.000	0.50	0
[0.002049,0.00247)	267	0.002	0.007	0.60	2
[0.002475,0.00300)	266	0.003	0.000	0.72	0
[0.002999,0.00388)	267	0.003	0.004	0.90	1
[0.003878,0.00542)	267	0.005	0.007	1.22	2
[0.005417,0.00860)	266	0.007	0.011	1.81	3
[0.008603,0.01736)	267	0.012	0.007	3.20	2
[0.017360,0.61830]	266	0.061	0.090	16.24	24

Based on EAM, CD4, w/o VL
Using 10 risk score bins; Hosmer-Lemeshow goodness of fit p-value: 0.1758
total obs.; total number of observations in risk decile
mean pred.; mean number of predicted viremic cases in risk decile
mean obs.; mean number of observed viremic cases in risk decile
pred. # cases; predicted number of viremic cases in risk decile
obs. # cases; observed number of viremic cases in risk decile

References

[1] S. B. McKearnan, J. Wolfson, D. M. Vock, et al. "Performance of the Net Reclassification for Nonnested Models and a Novel Percentile-Based Alternative". In: *American Journal of Epidemiology* (2018).
